# Treatment-Responsive Granulomatous-Lymphocytic Interstitial Lung Disease in a Pediatric Case of Common Variable Immunodeficiency

**DOI:** 10.3389/fped.2019.00105

**Published:** 2019-03-29

**Authors:** Robert Tillman, R. Paul Guillerman, Timothy Trojan, Manuel Silva-Carmona, Ivan K. Chinn

**Affiliations:** ^1^Pediatric Pulmonary, Baylor College of Medicine, Texas Children's Hospital, Houston, TX, United States; ^2^Pediatric Radiology, Baylor College of Medicine, Texas Children's Hospital, Houston, TX, United States; ^3^Allergy Immunology, Allergy Partners of Oklahoma, Endid, OK, United States; ^4^Pediatric Critical Care, Baylor College of Medicine, Texas Children's Hospital, Houston, TX, United States; ^5^Pediatric Allergy and Immunology, Baylor College of Medicine, Texas Children's Hospital, Houston, TX, United States

**Keywords:** Granulomatous-Lymphocytic Interstitial Lung disease, Common Variable Immunodeficiency, chemotherapeutic agents, pediatrics, treatment

## Abstract

Granulomatous-Lymphocytic Interstitial Lung disease (GLILD) is a granulomatous and lymphoproliferative condition occurring in ~25% of Common Variable Immunodeficiency (CVID) patients with the highest prevalence in the late teen to young adult years. GLILD was first described in adults and carries a poor prognosis with survival estimated to be reduced by half. Here we report a pediatric case of CVID-associated GLILD that presented with rapid deterioration over 3 months and responded to adult-based treatment with dual chemotherapeutic agents (rituximab and azathioprine), resulting in complete resolution of clinical findings and near complete resolution of radiologic findings. This case highlights the opportunity to achieve a favorable outcome in GLILD following appropriate diagnosis and therapy.

## Introduction

Common Variable Immunodeficiency (CVID) is the second most common primary immunodeficiency and is characterized by low antibody concentration with poor or absent response to immunization (1). Patients with CVID carry high risk for recurrent infections, lymphoproliferative disease, and autoimmune disorders. The majority of presenting symptoms are related to the respiratory tract with bronchitis, sinusitis, pneumonia, and bronchiectasis being the most common (2). A study of adults with CVID showed chronic structural lung abnormalities on high resolution chest CT scans in 73% (3). A pediatric study by van de Ven et al. showed structural lung abnormalities in 85–93%, although they were noted to be milder (4). Another study revealed that 10% of pediatric CVID patients as young as 2–5 years old had abnormalities in chest CT scans (5).

Pulmonary infections related to CVID can be primarily bacterial, fungal or viral, and, when recurrent, can result in bronchiectasis (6). Adequate intravenous immunoglobulin (IgG) replacement significantly reduces the infectious pulmonary complications of CVID (7). Noninfectious pulmonary complications are highly prevalent, occurring in 58% of adults with CVID (8). Noninfectious pulmonary complications include interstitial lung disease, asthma, and lymphoproliferative disorders.

Granulomatous-Lymphocytic Interstitial Lung disease (GLILD) is a pulmonary granulomatous and/or lymphoproliferative complication of CVID first described in adults and carries a poor prognosis. It was initially reported in 2004 by Bates et al. as part of a retrospective review of 69 patients. They showed that CVID patients without GLILD survived a median of 28.8 years after diagnosis of CVID, compared to 13.7 years for those with GLILD (8). GLILD has been estimated to occur in about 25% of the CVID population with a higher prevalence in the late adolescent to young adult age range (1).

Here we describe the presentation, clinical course, and treatment of GLILD complicating a pediatric case of GLILD. Written informed consent was obtained from the parents of the patient for the hospitalization and treatment of the patient, as well as for the publication of this case report and any potentially-identifying information.

## Case Report

**Figure 1:**
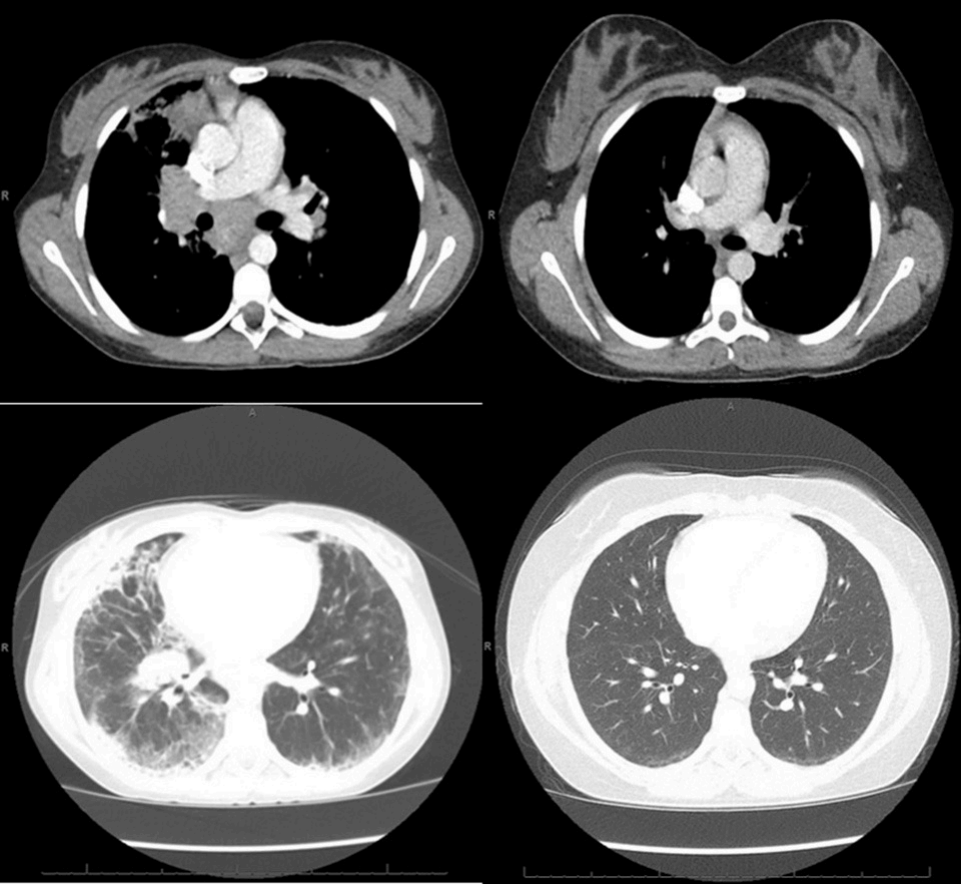
**Left**: CT of the chest obtained around the time of clinical presentation of GLILD, showing mediastinal and hilar lymphadenopathy, peripheral interlobular septal thickening, peripheral consolidation, and ground glass opacities, more prominent on the right lung than left. **Right**: CT of the chest obtained after treatment with 4 doses of rituximab and azathioprine showing resolution of the lymphadenopathy, consolidation and septal thickening and near resolution of the ground glass opacities.

**Figure 2:**
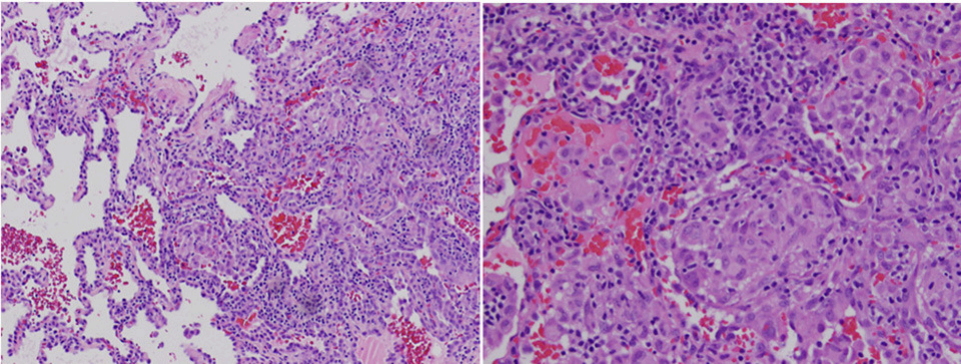
Right lower lobe lung wedge biopsy using a hematoxylin and eosin stain showing granulomatous lymphocytic Interstitial lung disease with non-caseating granulomatous inflammation with aggregates of small lymphocytes, scattered multinucleated giant cells, scattered foci of organizing pneumonia, interstitial fibrosis focally in the subpleural space but not prominent or diffuse, and airway luminal compromise from adjacent lymphoid hyperplasia.

A 13-year-old girl diagnosed the previous year with CVID in the setting of pneumonia, low serum immunoglobulin levels, and absent antibody responses to immunizations, presented with worsening dyspnea. Whole exome sequencing did not reveal an underlying genetic explanation for the immune deficiency. A chest CT scan at the time of CVID diagnosis revealed only mediastinal lymphadenopathy. She was placed on monthly IgG replacement therapy and did well until she reported gradual worsening of dyspnea over 3 months. The dyspnea first manifested during competitive sports and progressed to an inability to walk up a single flight of stairs. Pulmonary function testing results (Table 1) showed a restrictive pattern, and she was unable to complete the maneuvers for diffusing capacity of the lungs for carbon monoxide (DLCO). A follow-up chest CT scan revealed mediastinal and hilar lymphadenopathy, peripheral interlobular septal thickening, peripheral consolidation, and ground glass opacities (Figure 1). Bronchoalveolar lavage obtained by bronchoscopy did not show any evidence of infection. Specific testing included bacterial, fungal, mycobacterial, and viral cultures along with PCR assays for influenza, respiratory syncytial virus, parainfluenza, human metapneumovirus, adenovirus, cytomegalovirus, Epstein-Barr virus, human herpes virus-8, and *Pneumocystis jirovecii*. She underwent a right lower lobe wedge resection biopsy via thoracoscopy. The biopsy (Figure 2) showed non-caseating granulomatous inflammation with aggregates of small lymphocytes, scattered multinucleated giant cells, scattered foci of organizing pneumonia, interstitial fibrosis focally in the subpleural space but not prominent or diffuse, and airway luminal compromise from adjacent lymphoid hyperplasia, confirming the diagnosis of GLILD.

**Table 1:** Pulmonary Function Testing over the course of illness and treatment.

	**Presentation (%)**	**Two doses of rituximab (%)**	**Three doses of rituximab (%)**	**Four doses of rituximab (%)**	**1-year post therapy (%)**
FVC predicted	60	74	105	118	124
FEV1 predicted	64	74	101	110	113
TLC predicted	61	81	118	108	104
DLCO	N/A	78	83	76	99

*She was unable to complete DLCO at time of presentation due to symptoms. All values are given in percent predicted and the DLCO is corrected for both hemoglobin and alveolar volume. FVC, Forced Vital Capacity; FEV1, forced expiratory volume in 1 s; TLC, Total Lung Capacity; DLCO, diffusing capacity of the lungs for carbon monoxide*.

She was treated with 4 doses of rituximab 375 mg/m^2^ given 4–6 months apart, based on clinical symptoms and pulmonary function testing, and azathioprine 50 mg daily for 18 months as IgG replacement therapy was continued. She responded well to the new therapy regimen with complete resolution of exercise intolerance and normalization of pulmonary function testing parameters (Table 1). She also had remarkable improvement of the CT scan abnormalities (Figure 1) with the follow up CT scan after completion of therapy demonstrating resolution of all abnormalities.

## Discussion

A recent consensus statement of the British Lung Foundation/United Kingdom Primary Immunodeficiency Network (9) defined GLILD as a “distinct interstitial lung disease (ILD) occurring in patients with CVID, associated with a lymphocytic infiltrate and/or granuloma in the lung, and in whom other conditions have been considered and where possible excluded.” Diagnosis of GLILD in CVID is challenging and requires a high index of suspicion. It was first described by Bates et al. (8) with presenting findings including dyspnea, splenomegaly, pulmonary function testing with a restrictive pattern, and a DLCO that is usually low but sometimes normal (8). Hartono et al. found that splenomegaly, a history of idiopathic thrombocytopenia and autoimmune hemolytic anemia, and low level of immunoglobulin A increase the risk of developing GLILD (10). Our patient had dyspnea, restrictive lung function, splenomegaly and low concentration of immunoglobulin A at presentation. However, GLILD may be asymptomatic, and chest CT is important in the diagnostic evaluation (9).

In the initial description of GLILD by Bates et al. (8) the most common chest CT scan findings associated with GLILD included pulmonary consolidation, ground glass opacities, reticular opacities (septal thickening), and nodules. In the consensus statement of the British Lung Foundation/United Kingdom Primary Immunodeficiency Network (9), no chest CT findings were consistently rated as necessary to make the diagnosis, although pulmonary nodules, ground glass opacities, and lymphadenopathy were considered typical. The increased rate of infections in this patient population that can also lead to nonspecific CT scan findings further complicates the use of this radiologic technique to diagnose GLILD. For example, ground glass opacities frequently appear with viral infections and nodules with fungal infections. Our patient exhibited chest CT findings consisting of pulmonary consolidation, ground glass opacities, septal thickening, and lymphadenopathy, but no pulmonary nodules (Figure 1). It is unclear why pulmonary nodules were not visualized in our patient by chest CT, since biopsy revealed granulomatous inflammation and lymphocyte aggregates, either of which could manifest as nodules in a CT scan.

GLILD suspected on the basis of CT scanning requires a lung biopsy to confirm the diagnosis and to exclude the differential diagnostic considerations of infection, organizing pneumonia, sarcoidosis, lymphoid interstitial pneumonia, and lymphoma (9). The original report of GLILD described granulomatous disease with lymphocytic interstitial pneumonia, follicular bronchiolitis, and lymphoid hyperplasia on pathologic examination (8), and the granulomatous lung disease did not show the typical tight well-defined clusters of multinucleated giant cells but instead loose clusters of epithelioid cells, multinucleated giant cells and lymphocytes. A subsequent review of 16 confirmed adult cases (11) showed greater pathologic variety, including heterogeneous findings of nodular parabronchial inflammation of mature appearing lymphocytes, dense interstitial chronic infiltration, granulomata that were well to poorly formed, non-necrotizing epithelioid histocytes with occasional giant multinucleated cells, and also areas of fibrosis in some patients. This study revealed that the variety of pathologic findings depended upon the stage of illness. In the consensus statement of the British Lung Foundation/United Kingdom Primary Immunodeficiency Network (9), no pathologic features were consistently rated as necessary to make the diagnosis, although the presence of granulomatous inflammation, peribronchial and interstitial lymphoid proliferation, and CD4^+^ cell predominance were considered sufficiently typical to make a confident diagnosis in a patient with a primary immunodeficiency disease.

In patients with primary immunodeficiency disease who develop GLILD or other types of ILD, identification of a specific molecular diagnosis through whole exome sequencing or other methods can facilitate directed therapy (12). For example, patients with *LRBA* deficiency and *CTLA4* haploinsufficiency can develop GLILD and respond well to abatacept treatment (13–16). On the other hand, patients with underlying chronic granulomatous disease or defects in *RAG1* or *RAG2* may require hematopoietic stem cell transplantation (17–19). GLILD can also be observed in patients with thymic defects, such as partial DiGeorge anomaly and Good syndrome (20, 21). These conditions remain important to diagnose because of other associated complications. Interestingly, treatment of GLILD in *XIAP* deficiency has been reported with similar use of rituximab and azathioprine (22). The majority of patients, however, consist of adults who have CVID and no molecular diagnosis.

As noted above, previous studies in adults indicate increased mortality in patients with GLILD, emphasizing the need for developing an effective treatment in this population. The optimal treatment for GLILD remains unclear, and various therapy regimens have been suggested. The consensus statement of the British Lung Foundation/United Kingdom Primary Immunodeficiency Network recommends optimizing immunoglobulin therapy and then corticosteroids as first line treatment, with all other therapies considered on a case by case basis (9). In a small study on seven adult patients (23), treatment with rituximab and azathioprine resulted in improved pulmonary function testing and improved findings on follow-up CT, similar to that seen in our patient. The longer patients were treated with steroids before chemotherapeutics, the less they responded to the chemotherapeutics. Based on this time-related response, they theorized the time to diagnosis and effective treatment was directly related to the outcome.

Treatment for GLILD in CVID has chiefly been described in the older teenage and adult populations. One case has been reported at the time of publication of a 4-month-old with GLILD based on biopsy but no diagnosis of CVID who responded to immunoglobulin therapy (24). We used a combination of immune modulating agents that had previously demonstrated efficacy in a limited number of adult patients with CVID and GLILD. From a mechanistic perspective, lung biopsies from patients with GLILD show infiltrates containing T cells and B cells. Rituximab is directed toward B cells, and azathioprine suppresses T cells. We found that this combination therapy led to both subjective and objective resolution of GLILD in this pediatric patient.

Our patient received 4 rounds of rituximab and 18 months of azathioprine based on previously reported studies, but currently no data supports duration of treatment other than based upon symptoms. Treatment response should be assessed using symptoms, lung function, and imaging (9). Insufficient data are similarly available concerning need for maintenance therapy or routine scheduled follow up unless symptoms return. Our patient was followed until the end of therapy at our center and has now transitioned back to her home physicians. She has continued to do well off of rituximab and azathioprine with no return of symptoms. Her follow up CT scan at 1 year after completion of rituximab and azathioprine showed no abnormalities present and her repeat PFTs are normal (Table 1).

While likely requiring a multicenter approach, studies are still needed to compare steroids to other chemotherapeutic agents and trials are necessary even between single vs. dual agents. Further research is also required to determine length of therapy and need for long term follow up, including the potential need for maintenance medications outside of traditional CVID therapy. Based on our experience with this case, we believe that aggressive work up, rapid diagnosis, and aggressive treatment with the dual chemotherapeutic agents provides a good outcome in the pediatric population.

## Author Contributions

RT provided the initial manuscript and organization of edits from other authors as provided. IKC and RG provided multiple reviews and edits of the manuscripts thought the process. TT provided reviews and edits along with the updates and clinical status since being discharged from the treating institution. MS-C and IKC provided senior mentorship, oversight of the project and reviews and edits though the entire process. All authors approved the final manuscript as submitted.

### Conflict of Interest Statement

MS-C and IKC receive royalties from UpToDate. RT currently receives salary support via a grant funding support a portion of his salary from the North American Cystic Fibrosis Foundation Therapeutic Network (Grant MELICO17A0). These funds were not used as part of this paper. The remaining authors declare that the research was conducted in the absence of any commercial or financial relationships that could be construed as a potential conflict of interest.
